# Ocular Headaches

**Published:** 1894-07

**Authors:** Charles F. Stacey

**Affiliations:** Boston


### OCULAR HEADACHES.[1]

BY CHARLES F. STACEY, M.D., BOSTON.

Mr. President and Gentlemen,—It was with much pleasure I accepted your kind invitation to read a paper before you to-night, and truly appreciate the honor. The subject which I have chosen may not seem a very important one, but when you stop and consider the large number of people troubled with this painful affection, who often go many years, and perhaps a lifetime, with only temporary relief, and that by continual use of drugs, which soon lose their effectiveness, and at the same time leave more or less of a bad result on the general system, it seems to me, as eye-strain is the principal factor in such a large proportion of cases of functional headache, or migraine, that it should be worthy of more than a passing thought.

---

[1] Read before the American Academy of Dental Science, Boston, March 7, 1894.

I cannot agree with Lauder Brunton, who declares that the most common causes of headache are decayed teeth and irregularities of vision. But I recognize the fact that the proper treatment of decayed teeth may be the only relief in some cases of headache, whereas defects in vision are the direct cause in a very great number.

It is not to be wondered at that the statement of one writer, that all migraine was due to eye-strain, was received with decided opposition by careful observers; but during the last few years, by the combined efforts of oculists and neurologists, eye-strain has come to the front as a most important factor in this troublesome affection.

In Pepper's "System of Medicine," the article on Cephalalgia, or Headache, by Wharton Sinkler, under Sympathetic Headache, reads, "The headache from eye-strain may be considered in this connection, and deserves careful consideration. Many people have suffered from headache for years from this cause without its being suspected."

Weir Mitchell, in 1876, brought prominently to notice the frequency with which headache may be caused by defects of vision, and later, in 1891, he again called attention to this important subject in a very able paper.

Previous to this the general practitioner seldom recognized eye-strain as a cause of headache. It is now becoming a very common occurrence for the family doctor to refer cases of persistent headache, where the cause seems obscure, to the oculist, for examination of the refraction of the eye; and out of such cases I do not think I overstate it in saying that seventy-five per cent. or more are entirely, or to a very great extent, relieved from headache.

One of the most important facts in consideration of ocular headache is the pain. It has all degrees of severity; it may be more or less constant, or interrupted; it may be associated with the use of the eyes in reading, writing, or sewing, or any near work; also, from attending church, theatre, or concert, or from excessive mental or manual labor. The regions most frequently complained of are frontal, occipital, post-ocular, parietal, throughout the head, and in exceptional cases in the neck, arms, and shoulders.

Bright light, noises, locomotion, worry, or any mental strain, odors (as tobacco), usually increase the suffering. The most comfortable condition for the patient seems lying down in a darkened room with cooling lotions on the head, and perfect mental inactivity. But the best and most effective treatment for permanent relief is correction of the eye-strain by proper glasses.

Ocular headaches are very often not associated with poor sight. It is the rule that small errors of refraction, where the sight is exceptionally good, are often the cause of most troublesome symptoms; whereas the refractive error may be very large and the sight very poor, yet the symptoms may not be at all annoying. Thus it is not to be supposed that people whose eyesight is perfect, as far as they can judge, will be easily prevailed upon to adopt glasses as a curative measure in cases of headache.

Another fact must be mentioned: the eye-strain may remain latent for years and cause no troublesome symptoms, when by sickness or poor health, or an increased sensitiveness of the brain, either from mental or moral causes, it may make itself suddenly apparent.

The primary defects of the eye that cause derangements elsewhere are essentially functional in origin and result. They are principally errors of refraction and accommodation, classed under the name of Ametropia, or defective sight, and include astigmatism; hyperopia, or far-sightedness; myopia, or near-sightedness, separately or in combination.

The results of the above defects in the eye are called eye-strain.

J. J. Chisholm considers that astigmatism is a very common cause of headache, and that the class of persons in which it occurs are healthy people,—young, active, and industrious. They all suffer more or less with eye-pain and headache, which they call neuralgia, —a term that they have learned from their family doctor.

Mittendorf studied one thousand cases of ocular headache. He found an unusually large number of school children and college students, as well as women, who have had but little out-door exercise, and whose muscular system was undeveloped, were among his patients. He lays much stress on out-door exercise and gymnastics, as well as glasses, in the treatment of these cases.

Out of his one thousand cases the causes were: Astigmatism, eighty-three per cent.; hyperopia, or far-sightedness, eleven per cent.; myopia, or near-sightedness, two per cent. One hundred and nineteen were near-sighted astigmatism, seven hundred were far-sighted astigmatism.

Two hundred cases of ocular headache taken from my own records agree very closely with Mittendorf's report.

In my cases the causes were: Astigmatism, ninety per cent.; hyperopia, eight per cent.; myopia, two per cent. Of these, ninety per cent. received marked or entire relief from headaches by the aid of glasses.

Time will permit me to consider only briefly the three principal causes of ocular headache, taking them in the order of least importance.

### MYOPIA, OR NEAR-SIGHTEDNESS.

This causes the smallest percentage of ocular headaches, because in this defect in the sight the eye-strain comes in distant vision; and unless a man leads a life different from the great majority, his eyes are used three-quarters of the time for near work, and he often is satisfied with poor sight for distance, the strain in looking off being unnoticed. There are two kinds of near-sightedness:

*First.*—Elongation of the optical axis, the eye being too long from before backward.

*Second.*—Increased curvature of the lens or cornea.

It is congenital sometimes, especially among the poor. A great many children develop it after twelve or fifteen years. Eight per cent. of the community are estimated as being myopic. It is caused in many cases by unfavorable conditions at school, such as room, light, attitude of the head, distance of the book or paper, and duration of study.

Myopic people are, as a rule, easily embarrassed, and more fond of books than society; given to sedentary habits rather than outdoor, active pursuits. Their poor sight cannot be cured, but can be corrected by the adoption of properly-fitted concave or minus glasses for constant use.

### HYPEROPIA, OR FAR-SIGHTEDNESS.

This causes about one-tenth of all ocular headaches. In this affection, the opposite of myopia in most respects, the sight is very good for distance, but apt to blur for near. The eye-strain is continual, for the ciliary muscle tries to overcome the error, when the eyes are focussed for near, as well as distant vision.

The eye is far-sighted from—

*First.*—Shortening of the optical axis, the eye being too short.

*Second.*—Flattening of the lens or cornea.

Far-sighted people are often characterized by narrow faces, shallowed orbits, and eyes deep-set. The symptoms vary in this affection from none at all to severe ocular headache, and inability to read any length of time (in people under forty years) on account of the print blurring or running together. Glasses ordered oftentimes become indispensable, or may at first be used all the time, then later used only for near work.

The comfort of the patient is the fundamental reason for constant use of glasses, and also in very sensitive people, whose eyes give them much pain,—as semi-invalids, impressible and neuralgic patients,—while the dull, torpid, and unobservant will often be quite indifferent to the aid of glasses, even though they have a marked error.

To correct this affection we order convex or plus glasses.

### ASTIGMATISM.

This, as has been shown, is the error which causes four-fifths or more of ocular headaches. People in general have a very confused idea of astigmatism. It is an unequal curvature of the cornea or lens, or both. Instead of being spheres, they have a greater curvature in one direction than the other.

To use a rough illustration, we might say, the curvature of the cornea or lens in this affection is something like the outer surface of a spoon.

Now, in order to see clearly, the ciliary muscle has to contract irregularly, and in trying to accomplish this we get the eye-strain, which causes such a large number of severe and painful ocular headaches.

There are two kinds of astigmatism, regular and irregular. The latter is caused by disease of the cornea, and does not admit of satisfactory correction by glasses; the former, due to abnormal curvature of the cornea or lens, is corrected by cylindric or sphero-cylindric glasses.

Of correctable astigmatisms the greater number are congenital, and yet the error may not show itself during youth. A small amount of astigmatism is claimed to be natural to every one; but I cannot agree with such statements.

We have five forms of astigmatism, namely, far-sighted and compound far-sighted, near-sighted and compound near-sighted, together with mixed astigmatism.

In far-sighted astigmatism the defective axis is commonly the vertical, while in near-sighted it is the horizontal.

In mixed astigmatism one meridian is far-sighted and another is near-sighted.

The symptoms are severe ocular headaches, indistinct vision, according to the amount; also, in some cases, a person may notice a difference in the distinction of objects, as to their form.

In one case a patient could see the telegraph wire farther than he could see the pole on which it was stretched. Some patients cannot tell the time on a clock when the hands are in certain positions. College students have much trouble in distinctly seeing Greek, Hebrew, and German. Books are often held close to the eyes, and such patients are supposed to be near-sighted.

Want of quick perception is also a characteristic of this affection. Moreover, if the error be far- or near-sighted astigmatism, with the defective axis nearly horizontal in both cases, lines perpendicular are most distinctly seen. Persons with notable error get on moderately well, because most objects which we deal with have greater height than breadth. Such is the case with Roman type, trees, men (as a rule), buildings (especially in Chicago), statues, and the majority of objects. The height is often exaggerated, but the patient is not aware of this.

But if the axis is in the vertical or oblique direction, or the eyes are unsymmetrical, trouble announces itself early. An attack of illness, constant eye-work, excessive grief, etc., will reveal astigmatic errors which were previously unsuspected.

The more pronounced the head symptoms, the more searching must be the examination for any defects in sight, and if an error is found it should be immediately corrected by accurately-fitted glasses.

More glasses are worn now, not because people are not born with as good sight as they used to be years ago, but because the eyes are taxed more to-day than in the past.

Now, take your own profession, for instance. I think you will bear me out in the statement, that owing to the great advance in dentistry (especially operative), more time is required, and the eyes undergo a much greater strain in the details of the work than they did fifteen or twenty years ago.

This increased demand on our eyesight is seen also in all walks of life. Who would have supposed, twenty years ago, Sunday papers would ever print fifty to sixty pages or more, to be read on a day set apart for rest.

Another reason for glasses is because the eye has been more intelligently studied, and to-day many people are adopting glasses because they have proved their usefulness in the treatment of ocular headaches.

I will not go as far as the President of the Ophthalmic Section of the British Medical Association does, "who expresses the hope that the time will come when a man who goes about with his eyes naked will be so rare that the sight of him will almost raise a blush." But I will say that every person troubled with headache not traceable to some disturbance of the general system, who has not tried the use of appropriate glasses for relief, has neglected a most efficient cure furnished by optical therapeutics.

